# Wenyang Huazhuo formula extract ameliorates diabetic kidney disease in db/db mice and is associated with modulation of MHC class II molecules and gut microbiota

**DOI:** 10.3389/fphar.2026.1798497

**Published:** 2026-03-31

**Authors:** Qi Gao, Xingyao Li, Yanhong Zhao, Tiantian Jiang, Yuetong Wang, Hailong Zhang, Xili Wu

**Affiliations:** 1 Department of Traditional Chinese Medicine, The Second Affiliated Hospital of Xi’an Jiaotong University, Xi’an, China; 2 School of Pharmacy, Xi’an Jiaotong University, Xi’an, China

**Keywords:** db/db mice, DKD, gut microbiota, MHC class II, WYHZF extract

## Abstract

The purpose of this study was to investigate the therapeutic effects and underlying mechanisms of Wenyang Huazhuo Formula (WYHZF) extract in diabetic kidney disease (DKD) in db/db mice. DKD mice were administered WYHZF extract by oral gavage, and glycolipid metabolism, renal function, and renal pathology were assessed. Renal histopathology was evaluated using H&E, PAS, and Masson’s trichrome staining. UPLC-QE-Orbitrap-MS, HPLC, and UV-Vis spectroscopy were employed to characterize prototype and potential blood-absorbed metabolites of WYHZF extract. Renal RNA-seq was conducted to identify differentially expressed genes and related pathways. Molecular docking and molecular dynamics simulations were used as supportive *in silico* analyses to assess the plausibility of interactions between representative metabolites and H2-Aa/H2-Ab1. Western blotting (WB) was performed to validate H2-Aa and H2-Ab1 regulation and to examine fibrosis-associated proteins TGF-β and α-SMA. Gut microbiota alterations were evaluated by 16S rRNA sequencing. WYHZF extract significantly improved hyperglycemia and dyslipidemia, reduced UACR, serum creatinine, and BUN, alleviated glomerular/tubular injury, and restored Nephrin and NGAL expression. Transcriptomic analysis indicated aberrant activation of MHC class II–related pathways in DKD kidneys, which was modulated after WYHZF treatment. Docking and molecular dynamics simulations suggested compatible binding modes and stable interaction patterns between several representative metabolites and H2-Aa/H2-Ab1 under simulated conditions. WB confirmed that H2-Aa and H2-Ab1 were upregulated in DKD and were reversed by WYHZF; notably, TGF-β and α-SMA were also reduced, consistent with attenuation of renal fibrotic features. In addition, WYHZF partially restored gut microbiota diversity and corrected key taxonomic imbalances. Collectively, WYHZF extract may exert multi-level benefits in DKD, accompanied by modulation of MHC class II-related molecules, reduced fibrosis-associated signals, and gut microbiota remodeling.

## 1 Introduction

Diabetic kidney disease (DKD) is one of the most common and severe microvascular complications of diabetes, as well as one of the leading causes of end-stage renal disease. Its occurrence and progression involve multiple pathophysiological processes, including glucose and lipid metabolism disorders, inflammatory responses, and renal fibrosis (Jha et al., 2024; Zhang et al., 2025). At present, clinical practice mainly relies on the comprehensive management of blood glucose, blood pressure and blood lipids, combined with the administration of drugs such as angiotensin-converting enzyme inhibitors/angiotensin II receptor blockers and sodium-glucose cotransporter 2 inhibitors to delay the progression of DKD (de Boer et al., 2022; Jha et al., 2024). However, the overall efficacy of these drugs remains limited, and long-term use is often accompanied by adverse reactions such as hypotension, electrolyte imbalance and urinary tract infections (Chang et al., 2025). Therefore, there is an urgent need to explore safer and more effective alternative or adjuvant therapeutic strategies.

Traditional Chinese medicine has accumulated rich clinical experience in the prevention and treatment of DKD. With its distinct characteristic of multi-metabolite, multi-target and multi-pathway synergistic action, it can simultaneously intervene in multiple key links of the disease network and exert a comprehensive therapeutic effect (Bai et al., 2025; He et al., 2025; Qi et al., 2024; Yang et al., 2025). Wenyang Huazhuo Formula (WYHZF) is an empirical formula developed under the guidance of the TCM theory of “kidney-yang deficiency and internal accumulation of turbid toxin”. Clinical observations have demonstrated that it exerts definite efficacy in improving renal function and reducing proteinuria in patients with DKD (Song et al., 2017). This formula is mainly composed of a combination of botanical drugs including Cassia Twig, Milkvetch Root, Processed Rhubarb, Processed Aconite Root, Corni Fructus, and Poria, exerting the synergistic effects of warming kidney-yang, resolving turbidity and detoxifying, activating blood circulation and promoting diuresis as a whole. Modern pharmacological studies have preliminarily indicated that the active metabolites in WYHZF (such as astragaloside IV, ferulic acid and geniposidic acid) may exert renoprotective effects through multiple pathways including anti-inflammation, hypoglycemic activity and anti-fibrosis (Li et al., 2024; Zhu et al., 2024; Zohny et al., 2025). However, systematic research on WYHZF remains insufficient at present. Its complete chemical profile, as well as the underlying mechanisms through which it exerts integrated regulation via multiple targets and pathways to ameliorate DKD, has not yet been fully elucidated. This has restricted its clinical popularization, application, and modern scientific interpretation to a certain extent.

In recent years, the rapid development of systems biology and multi-omics technologies has provided powerful tools for an in-depth elucidation of the complex mechanisms of botanical drug combinations intervene in diseases (Xie et al., 2025; Zhao et al., 2024). The integrated strategy has become the mainstream paradigm for investigating the mechanisms of action of these botanical drugs (Wang et al., 2022; Wang et al., 2024a; Zeng et al., 2023). First, techniques such as ultra-performance liquid chromatography-quadrupole-electrostatic field orbitrap-mass spectrometry (UPLC-QE-Orbitrap-MS) were used to systematically identify the chemical metabolites and the absorbed metabolites in the serum after administration, thereby clarifying its direct material basis. Subsequently, transcriptomics was applied to characterize the changes in gene expression profiles after drug intervention at the global transcriptional level, aiming to discover novel regulatory proteins. Finally, molecular docking, molecular dynamics simulations, and Western blotting (WB) can further clarify how these botanical drug combinations act on the major protein targets. In addition, apart from the direct binding of drug molecules to disease-related targets, these drugs can also exert disease-improving effects by regulating the structure of gut microbiota or the abundance of related bacterial genera (Hao et al., 2025; Yue et al., 2022). The combined application of these approaches provides a basis for verifying the holistic molecular mechanisms underlying the therapeutic effects of these botanical drug combinations.

In this study, spontaneous type 2 diabetic db/db mice were employed as the DKD model to systematically evaluate the interventional efficacy and underlying mechanisms of WYHZF against DKD. Techniques including UPLC-QE-Orbitrap-MS high performance liquid chromatography (HPLC) and ultraviolet (UV) were adopted to systematically identify the chemical profiles of WYHZF and the potential absorbed metabolites in serum after administration. Combined with techniques such as transcriptomics molecular docking, molecular dynamics simulations and WB, we explored the molecular mechanisms by which WYHZF intervenes in DKD via MHC class II-related molecules. On another level, 16S rRNA sequencing was performed to assess the effects of WYHZF on the gut microbiota structure of DKD model animals. In summary, this study could provide experimental evidence and methodological reference for its clinical application and the modernization research.

## 2 Materials and methods

### 2.1 WYHZF’s preparation

All crude botanical drugs were purchased from Xi’an Yurun Chinese medicine professional market and complied with the quality requirements of Chinese Pharmacopoeia. Each botanical drug was authenticated by Prof. Xili Wu from the second affiliated hospital of Xi’an jiaotong university. Voucher specimens were deposited at the Chinese medicine specimen museum at the second affiliated hospital of Xi’an jiaotong university. The information of botanical drugs, including complete Latin names, pharmaceutical names, Chinese names, dosage and geographical origin, is presented in Table 1.

**TABLE 1:** The Latin names, pharmaceutical names, Chinese names, dosage and geographical origin of botanical drugs.

Chinese name	English name	Scientific name	Weight (g)	Geographical origin
Guizhi	Cassia twig	*Cinnamomum cassia* presl	30	Guangxi, China
Heifupian	Processed aconite root	*Aconitum carmichaelii* debx	12	Sichuan, China
Huangqi	Milkvetch root	*Astragalus membranaceus* (fisch.) bunge	50	Neimenggu, China
Shudahuang	Processed rhubarb	*Rheum palmatum* L	10	Qinghai, China
Cheqianzi	Plantain seed	*Plantago asiatica* L	30	Sichuan, China
Zelan	Hiraute shiny bugleweed herb	*Lycopus lucidus *Turcz. Ex benth	10	Jiangsu, China
Fuling	Poria	*Wolfiporia cocos* (schwein.) ryvarden & gilb	15	Hubei, China
Chuanxiong	Szechuan lovage rhizome	*Ligusticum chuanxiong* hort	12	Sichuan, China
Jinyingzi	Cherokee rose fruit	*Rosa laevigata* michx	50	Jiangxi, China
Qianshi	Gordon euryale seed	*Euryale ferox* salisb. Ex konig et sims	30	Jiangsu, China
Shanzhuyu	Cornus fruit	*Cornus officinalis* sieb. et zucc	15	Henan, China

The 11 botanical drugs were mixed according to the prescription (Table 1), soaked in purified water for 30 min at room temperature using a solid-to-liquid ratio of 1: [10] (w/v). The soaked mixture was decocted under atmospheric pressure for 60 min. Decoction was performed in an extraction vessel with continuous mild boiling (approximately 95 °C–100 °C), and the mixture was gently stirred every 5 min to ensure uniform extraction. After decoction, the hot extract was immediately filtered through 200 mesh nylon filter to remove insoluble residues. The residue was by adding purified water at the same solid-to-liquid ratio and decocting for another 30 min, and the two filtrates were pooled. The combined filtrate was allowed to cool to room temperature and then clarified by centrifugation at 3,000 g for 20 min to minimize suspended particulates. The clarified decoction was concentrated under reduced pressure using a rotary evaporator (Re100 pro, Dalong, China) at 50 °C and −0.08 MPa until the extract reached a final concentration corresponding to 1 g crude drug/mL. The concentrate was frozen at −80 °C and subsequently lyophilized using a freeze-dryer (LGJ-100E, Sihuan, Beijing, China]) under 5.5 Pa, yielding a dry extract powder.

The extraction yield was approximately 4.5% (w/w) relative to the total crude-drug mass, calculated as: Yield (%) = (mass of dry extract powder/mass of total crude drugs) × 100%. The freeze-dried powder was stored at 4 °C in a sealed container protected from light and moisture. Before administration, the freeze-dried extract powder was freshly reconstituted in 0.9% sodium chloride to the target concentrations, vortexed until fully dispersed, and administered by oral gavage at a constant volume.

### 2.2 Methodology for UPLC-QE-orbitrap-MS, HPLC and UV-Vis analysis

Three orthogonal fingerprints were established for the WYHZF extract, including HPLC, UV-Vis spectrum (200–400 nm), and UPLC-QE-Orbitrap-MS. The HPLC chromatographic fingerprint and UV-Vis spectral fingerprint were primarily applied to characterize the chemical specificity of the WYHZF extract and to evaluate its batch-to-batch reproducibility.

UPLC-QE-Orbitrap-MS (Thermo Fisher Scientific, United States) was used for the non-targeted analysis of the prototype metabolites of WYHZF extract and the absorbed metabolites in serum, so as to comprehensively detect the chemical composition of the samples. For WYHZF extract, a certain amount of sample was filtered through a 0.22 μm microporous membrane to prepare the test solution; and for serum samples, they were deproteinized by adding 3-5 volumes of acetonitrile and centrifuging at 15,000 rpm for 15 min, after which the supernatant was dried under nitrogen, reconstituted in an appropriate volume of 50% acetonitrile, and filtered through a 0.22 μm microporous membrane to obtain the test solution. Chromatographic separation was performed on an Accquity™ BEH C18 column (3.0 mm × 100 mm, 2.6 μm) with the column temperature maintained at 30 °C. The mobile phase consisted of water containing 0.1% (v/v) formic acid (phase A) and acetonitrile (phase B), with an initial ratio of 95% A+ 5% B. The gradient elution program was as follows: 5%–10% B from 0 to 5 min; 10%–25% B from 5 to 20 min; 25%–95% B from 20 to 40 min. The flow rate of the mobile phase was 0.3 mL/min, and the injection volume was 5 μL.

The main purpose of the HPLC and UV-visible analyses was to evaluate the stability of different batches of WYHZF by examining their characteristic fingerprint profiles. All WYHZF samples were filtered through a 0.22 μm microporous membrane to obtain the test solutions. HPLC analysis was performed on a Welch ULtimate C18 column (4.6 × 250 mm, 5 μm). The mobile phase consisted of 0.1% phosphoric acid in water (A) and acetonitrile (B), with a gradient elution as follows: 0–3 min, 5% B; 3–10 min, 5%–20% B; 10–20 min, 20%–40% B; 20–25 min, 40%–60% B. The flow rate was set at 1.0 mL/min, the column temperature was maintained at 40 °C, the injection volume was 20 μL, and the detection wavelength was 254 nm. UV–Vis absorption spectra of different batches of WYHZF were recorded on a SpectraMax ABS Plus microplate spectrophotometer (Molecular Devices, United States). Test solutions were prepared as described above and 200 μL of each sample was added into a 96-well plate, with the corresponding solvent as blank. Full-wavelength scanning was performed in the range of 200–600 nm with a step size of 1 nm, and baseline-corrected spectra were exported as absorbance versus wavelength data. Inter-batch similarity of the HPLC and UV-Vis fingerprints was evaluated using Pearson correlation.

### 2.3 Animal experiment

All animal experimental procedures were strictly performed in accordance with the Guide for the Care and Use of Laboratory Animals published by the U.S. National Institutes of Health, and complied with the ARRIVE guidelines (Animal Research: Reporting *In Vivo* Experiments) to ensure the transparency and reproducibility of animal study reports. The protocol of this study has been approved by the Health Science Center of Xi’an Jiaotong University Approval for Research Involving Animals (Approval No.: XJTU.AE2025-3366).

All animals used in this study were purchased from SPF Biotechnology Co., Ltd. (Beijing, China). Eight-week-old pathogen-free male spontaneous type 2 diabetic db/db mice were selected for the intervention study of DKD, as these mice represent a classic animal model for such research. After 1 week of adaptive feeding, the fasting blood glucose (FBG) levels of the mice (following 6 h of fasting) were measured using a Roche Accu-Chek glucometer (Roche Diagnostics Products Ltd., Shanghai, China). Experimental intervention was initiated only after confirming that their FBG was at least 11.1 mmol/L. C57BL/6J mice were used as the experimental control group, and their fasting blood glucose levels were also measured prior to the experiment. After the detection, the db/db mice were randomly allocated to ensure that there were no significant differences in baseline indicators such as body weight and blood glucose among the groups.

The experimental intervention grouping and administration protocol were as follows: control group (C57BL/6J, n = 6), model group (db/db, n = 6), WYHZF_L group (db/db, WYHZF extract dose 0.45 g/kg/day, n = 6), WYHZF_H group (db/db, WYHZF extract dose 0.9 g/kg/day, n = 6), and valsartan group (db/db, 10 mg/kg/d, n = 6). The administered dosage was determined with reference to published studies on the intervention of relevant metabolites in DKD (Peng et al., 2023; Deng et al., 2025). Mice in the control group and model group were orally administered 0.9% sodium chloride solution; mice in the remaining groups received the corresponding drug treatments for eight consecutive weeks. All mice had free access to food and water, and were housed in a pathogen-free environment with the temperature maintained at 23 °C ± 1 °C, relative humidity at 50%–60%, and a 12-h light-dark cycle.

Two days prior to sacrifice, the mice were individually housed in metabolic cages (fasted but with free access to water) for the collection of 24-h urine samples, followed by measurements of body, kidney weight and FBG. After these procedures, the mice were placed in an anesthesia chamber and administered 3%–5% isoflurane (RWD Life Science Co., Ltd., Shenzhen, China) with oxygen as the carrier gas at a flow rate of 1–2 L/min. When the mice lost their righting reflex and exhibited stable breathing, the isoflurane concentration was maintained at 1%–2% to ensure the absence of pain response. Blood was collected into blood collection tubes by rapidly enucleating the mouse’s eyeball to drain the blood. Immediately after blood collection, the mice were euthanized via deep anesthesia combined with cervical dislocation. Subsequently, the pancreas, kidneys, and feces were harvested and preserved in fixative solution or liquid nitrogen for subsequent experiments.

### 2.4 Biochemical assay

Oral glucose tolerance test (OGTT) was performed on all mice prior to sacrifice: glucose was administered orally at a dose of 1 g/kg body weight. Blood samples were collected via tail snipping at designated time points (0, 15, 30, 60, 90, and 120 min), and blood glucose levels were measured using a glucometer (Wang et al., 2024b). The homeostasis model assessment of insulin resistance (HOMA-IR) was calculated using the formula: HOMA-IR = (fasting insulin level, FINS) x (fasting plasma glucose level, FPG)/22.5.

Serum creatinine (Scr), blood urea nitrogen (BUN), glycosylated hemoglobin A1c (GHbA1c), and insulin were measured using ELISA kits from Jiancheng Bioengineering Institute, Nanjing, China. Levels of total cholesterol (TC), triglycerides (TG), low density lipoprotein cholesterol (LDL-C), and high-density lipoprotein cholesterol (HDL-C) were determined using an automatic biochemistry analyzer (BS-200, Mindray Medical, Shenzhen, China). The urine albumin creatinine ratio (UACR) was detected using an automatic dry chemistry urine analyzer (UA-5600, Mindray, Shenzhen, China).

### 2.5 Histological staining

Immediately after harvesting, the pancreatic and renal tissues of mice were fixed in 4% paraformaldehyde solution at room temperature for 24–48 h, followed by routine paraffin embedding and sectioning. H&E staining: After dewaxing with xylene and hydration with gradient ethanol, the sections were stained with hematoxylin and eosin sequentially, then dehydrated, cleared, and mounted with neutral gum. The morphological changes of pancreatic islets and renal tissues were observed under a light microscope. PAS staining: Following dewaxing and hydration, renal tissue sections were stained using a PAS staining kit in accordance with the manufacturer’s instructions. This staining method was mainly used to observe and quantitatively analyze DKD-related pathological changes, such as glomerular basement membrane thickening and mesangial matrix proliferation. Masson staining: After Masson’s trichrome staining of renal tissue sections, collagen fibers (stained blue) and muscle fibers (stained red) were distinguished under a light microscope. This method was applied to evaluate the degree of tubulointerstitial and glomerular fibrosis.

The procedure of immunofluorescence was as follows: after dewaxing, hydration and antigen retrieval of renal paraffin sections, the sections were incubated with specific primary antibodies against Nephrin or NGAL at 4 °C overnight. On the following day, the corresponding fluorescence-labeled secondary antibodies were added for incubation. After nuclear staining with DAPI, the sections were sealed. Images were captured under a fluorescence microscope at the corresponding excitation/emission wavelengths, and the fluorescence intensities of Nephrin and NGAL were measured.

### 2.6 Transcriptome sequencing and analysis

Mouse renal tissues were harvested following standard procedures and immediately stored at −80 °C. Total RNA was extracted using the trizol method. After passing the quality control for concentration and integrity, mRNA was enriched with Oligo (dT) magnetic beads, fragmented under high-temperature conditions, and then used for double-stranded cDNA synthesis. End repair, poly (A) tail addition, and adapter ligation were performed sequentially to construct the sequencing library. After the library passed quantification and fragment length verification, paired-end sequencing was carried out on the Illumina NovaSeq 6,000 platform.

Raw sequencing data were subjected to quality control with FastQC and filtration with trimmomatic to obtain clean reads, which were then aligned to the mouse reference genome using HISAT2. Gene expression levels were calculated with StringTie/HTSeq. Subsequently, DESeq2 was used to screen for differentially expressed genes with the criteria of |log_2_FoldChange| ≥ 1 and P < 0.05. Principal component analysis (PCA) was performed to investigate differences in expression patterns among samples. Furthermore, Cluster Profiler was applied to conduct GO and KEGG enrichment analyses of differentially expressed genes (DEGs), so as to screen significantly enriched biological processes and signaling pathways.

### 2.7 Molecular docking and molecular dynamics analysis

We obtained 3D structures in SDF format via the PubChem database, then loaded them into ChemBio3D Ultra 14.0 to carry out energy minimization. The protein structures of H2-Aa (P01910) and H2-Ab1 (Q9CQ70) were retrieved from Uniprot; after eliminating crystal water from these proteins, we preprocessed the structures with Pymol2.3.0. Next, we conducted hydration, charge calculation, and charge assignment based on the optimized small-molecule structures, before importing the treated structures into AutoDock Vina (version 1.5.6). For the final step, we analyzed the docking outcomes using Pymol2.3.0 and LigPlot V2.2.8. A binding energy value of ≤ −5.0 kcal/mol was designated as a potentially effective binding interaction.

Molecular dynamics (MD) simulations were performed with GROMACS 2020.6 (www.gromacs.org). Using the AMBER99 force field and TIP3P water model, complex systems were constructed by combining H2-Aa and H2-Ab1 with each candidate ligand. These systems were placed in periodic water boxes, with an appropriate amount of Na^+^/Cl^−^ added to neutralize the overall charge. After undergoing energy minimization and NVT/NPT pre-equilibration, the systems were subjected to a 10 ns production simulation with a time step of 2 fs. Subsequently, indicators including RMSD, RMSF, radius of gyration (Rg), and hydrogen bond count were calculated, and the results were subjected to visual analysis to evaluate the conformational stability and binding durability of the protein-ligand complexes. In this study, we applied these two techniques to perform predictive interaction analysis between the key blood-absorbed components of WYHZF and H2-Aa and H2-Ab1.

### 2.8 WB verification

Mouse renal tissues were homogenized and lysed in RIPA lysis buffer containing protease inhibitors. The supernatant was collected, and the protein concentration was determined using a BCA protein quantification kit. Protein samples were mixed with 5x loading buffer and denatured at 95 °C for 5 min. Equal amounts of protein (20 μg) were subjected to SDS-PAGE electrophoresis and then transferred onto PVDF membranes. The PVDF membranes were blocked with TBST solution containing 5% BSA at room temperature for 1 h, followed by incubation with primary antibodies (H2-Aa, H2-Ab1, α-SMA, TGF-β, and the internal reference) at 4 °C overnight. On the following day, the membranes were washed with TBST and incubated with HRP-conjugated secondary antibodies. Band signals were detected via ECL chemiluminescence, and the gray value quantification of target bands was performed using image analysis software.

### 2.9 16S rRNA sequencing and analysis

Genomic DNA was extracted from fecal samples using a fecal DNA extraction kit (Cwbio, Jiangsu, China). The quality and concentration of the extracted DNA were evaluated via agarose gel electrophoresis and a Nanodrop® ND-2000 spectrophotometer (Thermo Scientific Inc., United States). The V3-V4 hypervariable regions of the 16S rRNA gene were amplified with universal primers. After purification and quantification of the PCR products, sequencing was performed on the Illumina NovaSeq 6,000 platform.

Data analysis was completed using the QIIME2 pipeline: DADA2 was employed to identify amplicon sequence variants (ASVs); taxonomic annotation was conducted using the VSEARCH algorithm combined with the Silva database. α-diversity indices (e.g., Shannon index, Chao1 index) were used to evaluate species richness and diversity within individual samples. β-diversity analyses based on principal coordinate analysis (PCoA) and non-metric multidimensional scaling (NMDS) were performed to compare the microbial community composition among different samples. Visualization was achieved via PCoA and volcano plots to identify microbial taxa with significant variations across experimental groups.

### 2.10 Data analysis

Numerical data were expressed as mean ± standard deviation (mean ± SD) and derived from at least 3 independent experiments. GraphPad Prism 9.0 software was used for statistical analysis to evaluate the significance of intergroup differences: one-way analysis of variance was applied for intergroup comparisons; Spearman’s correlation analysis was adopted for multivariate correlation analysis. A value of P < 0.05 was considered statistically significant. Significant differences among the normal control group, model group, and intervention groups were marked with asterisks: *P < 0.05, **P < 0.01, ***P < 0.001.

## 3 Results

### 3.1 Metabolite profiling of the WYHZF extract

**FIGURE 1:**
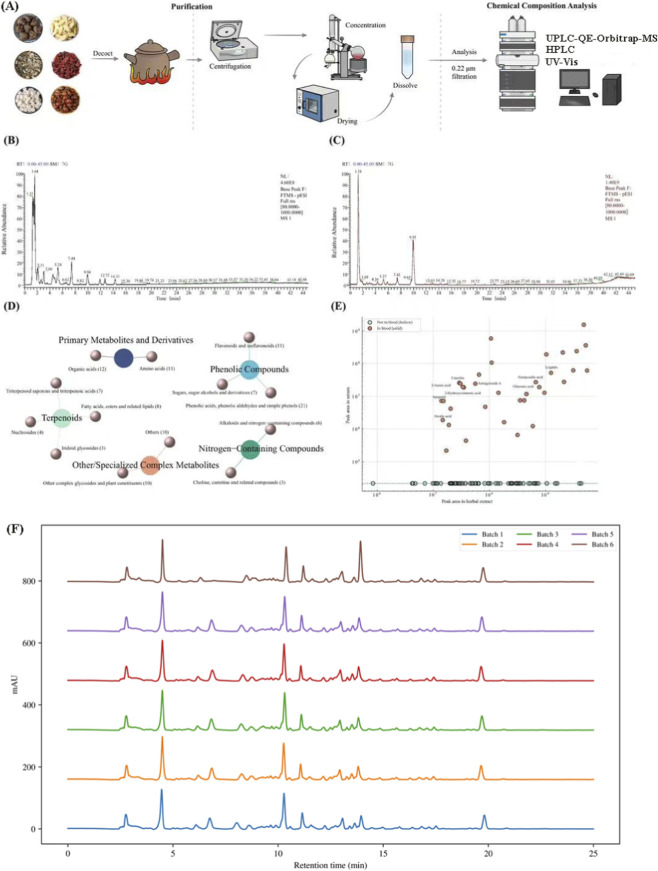
Metabolite profiling of the WYHZF extract. **(A)** Preparation and detection process; **(B)** Mass spectrometry data in positive ion mode; **(C)** Mass spectrometry data in negative ion mode; **(D)** Classification of chemical metabolites; **(E)** Prototype metabolites and potential blood-absorbed metabolites; **(F)** HPLC fingerprints of WYHZF extract.

WYHZF is composed of a total of 11 medicinal materials, namely, Cassia Twig (30 g), Processed Aconite Root (12 g), Milkvetch Root (50 g), Processed Rhubarb (10 g), Plantain Seed (30 g), Hiraute Shiny Bugleweed botanical drug (10 g), Poria (15 g), Szechuan Lovage Rhizome (12 g), Cherokee Rose Fruit (50 g), Gordon Euryale Seed (30 g), and Cornus Fruit (15 g) (Table 1). These medicinal materials are formulated in accordance with the “monarch, minister, assistant and guide” principle of Chinese medicine theory to exert a synergistic therapeutic effect optimally. WYHZF was prepared using the traditional decoction method, and the resulting decoction was subjected to centrifugation, concentration and drying for subsequent experiments (Figure 1A). It is worth noting that WYHZF is a multi-botanical drug and multi-metabolite system, and clarifying the information on its main chemical metabolites is a prerequisite for further investigating its therapeutic effects.

The UPLC-QE-Orbitrap-MS was applied to identify the chemical composition profile of WYHZF extract. The data-dependent acquisition mode with positive and negative ion switching was adopted to acquire the mass spectrometry information of the metabolites, respectively (Figures 1B,C). A total of 113 chemical metabolites were preliminarily identified and assigned to the respective medicinal materials of WYHZF extract (Supplementary Table S1). In addition to primary metabolites, these chemical metabolites were categorized into phenolic metabolites, terpenoids, nitrogen-containing metabolites, and other classes. Phenolic acids, phenolic aldehydes and simple phenols, flavonoids and isoflavonoids, and triterpenoid saponins and triterpenoic acids were the predominant metabolites (Figure 1D).

Furthermore, this study analyzed the serum chemical composition profile of mice following intragastric administration of the drug. Through comparative analysis with the prototype metabolites of WYHZF extract, a total of 36 metabolites were identified as potential absorbed metabolites (Figure 1E). Among these, the representative chemical metabolites included loganin and geniposidic acid derived from Cornus Fruit, astragaloside A and ononin derived from Milkvetch Root, ferulic acid derived from Szechuan Lovage Rhizome, gallic acid primarily derived from Cherokee Rose Fruit, gluconic acid derived from Poria, and 2-hydroxycinnamic acid derived from Cassia Twig (Supplementary Table S1).

In addition, to evaluate the inter-batch stability of WYHZF extracts, we performed similarity analysis of different batches using HPLC fingerprinting and UV-Vis scanning (Figure 1F; Supplementary Figure S1). The HPLC fingerprints showed highly consistent retention times and peak shapes for the common peaks, with inter-batch similarities calculated by a correlation-coefficient-based algorithm ranging from 0.83 to 0.99 (Supplementary Table S2). The UV-Vis spectra exhibited almost identical curve profiles and major absorption peaks in the 200–600 nm range, with similarities of 0.99–1.00 (Supplementary Table S3). These results indicate that WYHZF extracts possess stable and reproducible characteristic fingerprints, with only minor process variation among batches. Taken together with the aforementioned UPLC-QE-Orbitrap-MS-based systematic identification of prototype and absorbed metabolites, this study provides a relatively comprehensive characterization of the chemical profile of WYHZF extracts, thereby offering a reliable material basis for subsequent animal intervention and mechanistic studies.

### 3.2 Effects of WYHZF extract on glucose and lipid metabolism in db/db mice

**FIGURE 2:**
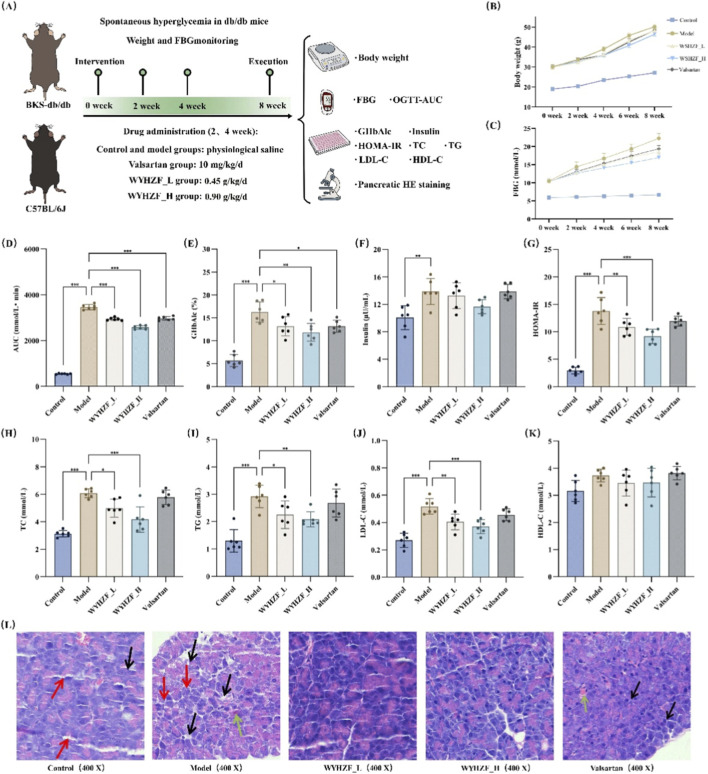
Effects of WYHZF on abnormal glucose and lipid metabolism in db/db mice. **(A)** Animal experiment and sampling protocol; **(B)** Changes in animal body weight; **(C)** Changes in FBG; **(D)** Comparison of AUC in OGTT; **(E)** Comparison of GHbA1c; **(F)** Comparison of insulin; **(G)** Comparison of HOMA-IR; **(H)** Comparison of TC; **(I)** Comparison of TG; **(J)** Comparison of LDL-C; **(K)** Comparison of HDL-C; **(L)** Staining of Pancreatic Tissues. (Note: * for P < 0.05, ** for P < 0.01, and *** for P < 0.001).

In this study, we first investigated the effects of WYHZF on abnormal glucose and lipid metabolism in db/db mice (Figure 2A). The body weights of mice in the model group and each administration group increased significantly over time and remained consistently higher than those in the normal control group, indicating that db/db mice exhibit an obese phenotype. In comparison with the model group, the body weights of mice in different administration groups showed a decrease (Figure 2B). Results of FBG showed that different administration groups reduced the blood glucose levels, with the high-dose WYHZF group exerting the optimal effect (Figure 2C). The area under the OGTT curve showed that the AUC of the model group was significantly higher than that of the control group, indicating a marked impairment of glucose tolerance. Both low- and high-dose WYHZF as well as valsartan significantly reduced the AUC and improved glucose tolerance (P < 0.001) (Figure 2D). The level of GHbA1c in the model group increased significantly, while treatment with different doses of WYHZF and valsartan notably decreased GHbA1c levels (P < 0.05) (Figure 2E). Meanwhile, compared with the control group, the model group exhibited hyperinsulinemia; the high-dose WYHZF reduced insulin levels (Figure 2F). The results of HOMA-IR assay demonstrated that the insulin resistance index of the model group was significantly elevated. After WYHZF treatment, the HOMA-IR index decreased significantly, suggesting that insulin resistance was alleviated (P < 0.01) (Figure 2G).

Figures 2H–K illustrate the characteristics of changes in the blood lipid profiles of the experimental animals. The results showed that the TC level in the model group was significantly higher than that in the control group; both low- and high-dose WYHZF treatments reduced TC levels, with the high-dose group exhibiting a more pronounced decrease (P < 0.05). The TG level in the model group increased significantly, whereas treatment with WYHZF at different doses resulted in a marked reduction in TG levels (P < 0.05). In addition, the LDL-C level was significantly elevated in the model group, and intragastric administration of WYHZF at different doses significantly lowered its level (P < 0.01). However, neither WYHZF nor valsartan intervention exerted a significant effect on HDL-C levels.

In the control group, pancreatic islets showed intact structures with distinct cell boundaries and regularly arranged acini. By contrast, islet cells in the model group were disordered, swollen and loosely arranged; partial acinar fragmentation (black arrows) and extensive cytoplasmic vacuolization (red arrows) were observed, indicating severe diabetes-associated pathological damage. Both low- and high-dose WYHZF treatment significantly alleviated these pathological changes, restoring relatively clear islet outlines and regular cell morphology. Valsartan treatment also improved islet structure compared with the model group, but mild abnormalities remained, such as pink amyloid deposits (green arrows) and slightly loose acinar arrangement (black arrows) (Figure 2L). Collectively, these results demonstrated that WYHZF could ameliorate pancreatic pathological damage in db/db mice. In summary, WYHZF can significantly ameliorate the abnormal glycolipid metabolism in db/db mice. The WYHZF_H group exhibited the optimal therapeutic effect, while valsartan exerted a relatively weak ameliorative effect.

### 3.3 Effects of WYHZF extract on renal function in db/db mice

**FIGURE 3:**
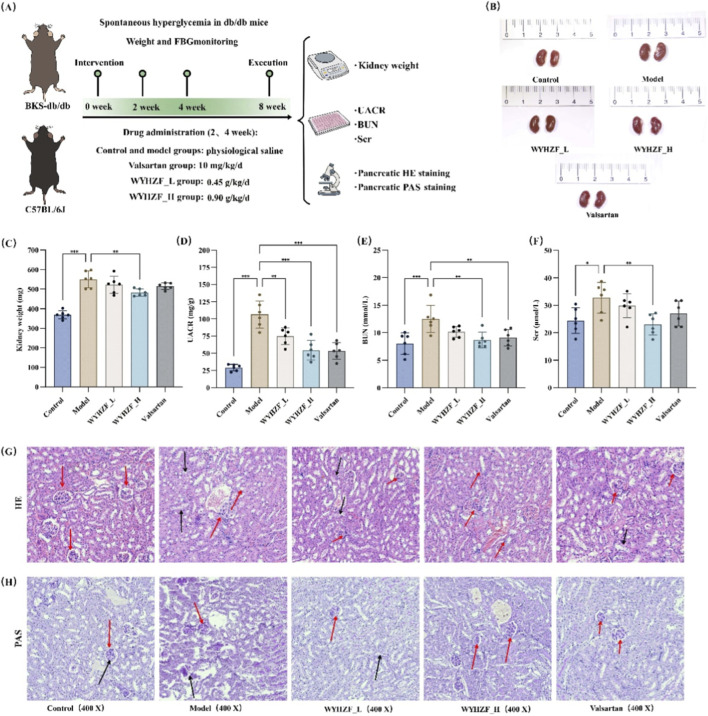
Effects of WYHZF on renal function in db/db mice **(A)** Animal experiment and sampling protocol, **(B)** Renal morphology, **(C)** Comparison of kidney weight, **(D)** Comparison of UACR, **(E)** Comparison of BUN, **(F)** Comparison of Scr, **(G)** HE Staining of renal tissues, **(H)** PAS staining of renal tissues. (Note: * for P < 0.05, ** for P < 0.01, and *** for P < 0.001).

To further clarify the protective effects of WYHZF against DKD in db/db mice, this study measured renal function and morphology-related indicators including kidney weight, UACR, BUN, and Scr. Combined with renal H&E staining and PAS staining, we comprehensively evaluated the effects of WYHZF on renal structure and function in DKD mice (Figure 3A).

Figures 3B,C illustrate the morphological and weight characteristics of renal tissues across different groups. Compared with the control group, the kidney weight of mice in the model group was significantly increased. After intervention, the kidney weight of mice was notably reduced, with the WYHZF_H group showing the most prominent weight-lowering effect. In the control group, UACR remained at a low level, while it was significantly elevated in the model group. Both the low- and high-dose WYHZF groups, as well as the valsartan group, significantly decreased UACR levels (P < 0.01) (Figure 3D). BUN and Scr levels of mice were significantly increased in the model group. After high-dose WYHZF intervention, the BUN and Scr levels were significantly reduced (P < 0.01), which was comparable to the improvement effect of the valsartan group (Figures 3E,F).

H&E staining showed that db/db model mice exhibited enlarged glomeruli with increased intraglomerular cells, obvious tubular epithelial vacuolization, and interstitial edema, inflammatory cell infiltration, and fibrosis (blue/black arrows). PAS staining further revealed marked mesangial expansion, increased mesangial matrix, thickened glomerular and tubular basement membranes, and abundant glycogen deposits in renal tubular epithelial cells. Low-dose WYHZF partially improved these lesions, whereas high-dose WYHZF markedly reduced glomerular mesangial hyperplasia, tubular injury, fibrosis, and glycogen deposition, with renal morphology approaching that of the positive control and normal groups (Figures 3G,H), indicating a clear protective effect on diabetic kidney injury. These results indicate that WYHZF effectively improves renal function and attenuates pathological renal injury in db/db mice with DKD.

### 3.4 Effects of WYHZF extract on renal injury markers in db/db mice

Nephrin is a key structural protein of the podocyte slit diaphragm, and its expression level directly reflects the integrity of the glomerular filtration barrier (Wada et al., 2016). NGAL is widely recognized as a sensitive marker for renal tubular injury and early renal impairment (Devarajan, 2010). Therefore, this study detected the expressions of Nephrin and NGAL in renal tissues using immunofluorescence technology to evaluate the interventional effect of WYHZF on DKD.

**FIGURE 4:**
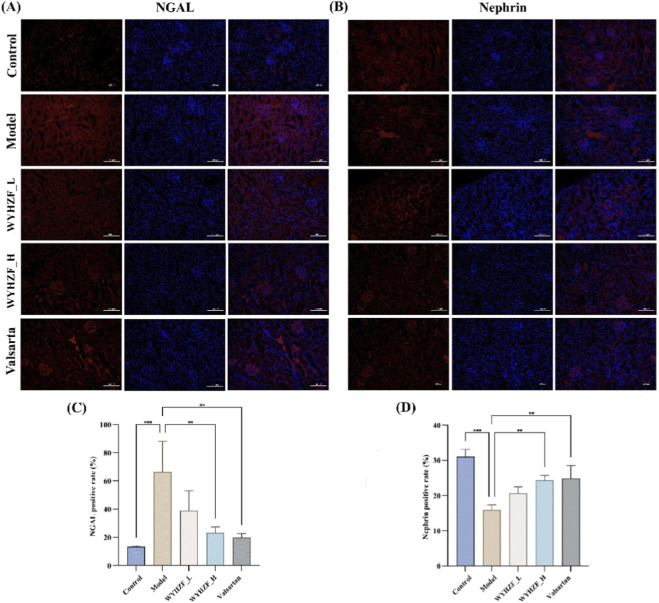
Effects of WYHZF on renal Injury markers in db/db mice. **(A)** Immunofluorescence results of NGAL in renal tissues; **(B)** Immunofluorescence Results of Nephrin in Renal Tissues; **(C)** Comparison of NGAL Positive Rate in Renal Tissues; **(D)** Comparison of Nephrin Positive Rate in Renal Tissues. (Note: * for P < 0.05, ** for P < 0.01, and *** for P < 0.001).

The results showed that the positive rate of NGAL in renal tissues of the model group was significantly higher than that of the control group, indicating that tubular injury and renal stress response were significantly aggravated under diabetic conditions. After WYHZF intervention, especially in the high-dose group, the positive rate of NGAL decreased significantly by more than 50%, approaching the level of the valsartan group (Figures 4A,B). In contrast, the positive rate of Nephrin in glomeruli of the model group was significantly lower than that of the control group, suggesting impairment of the filtration barrier. Following WYHZF treatment, the positive rate of Nephrin increased significantly, with the elevation amplitude in the high-dose group being comparable to that in the valsartan group (Figures 4C,D). These results further confirmed that this metabolite exerts a favorable overall interventional effect and renal protective role in db/db mice with DKD.

### 3.5 Effects of WYHZF extract on the renal transcriptome profile in db/db mice

**FIGURE 5:**
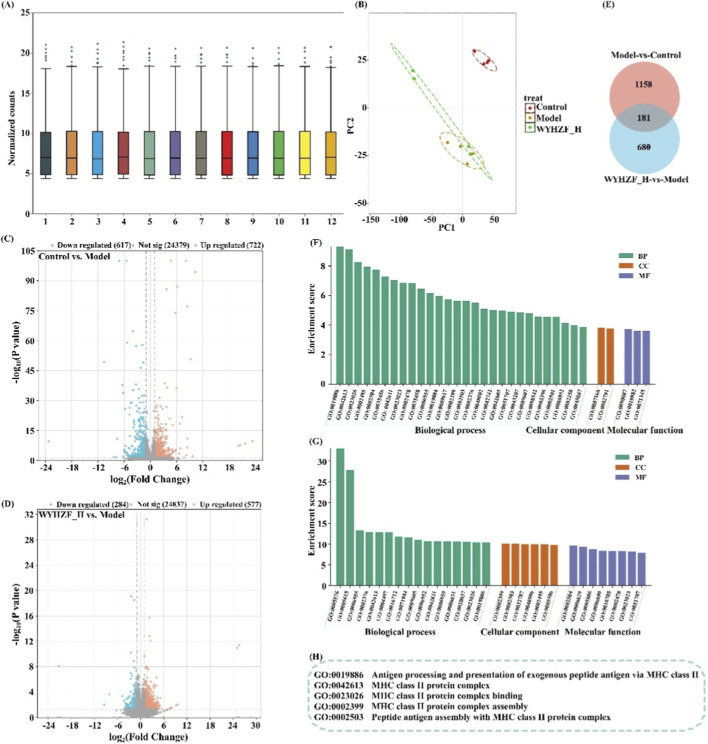
Effects of WYHZF on the renal transcriptome profile in db/db mice. **(A)** Boxplots of normalized gene expression levels; **(B)** PCA analysis; **(C)** Venn diagram analysis results; **(D)** Volcano plot of the normal group between model group; **(E)** Volcano plot of the WYHZF_H group between model group; **(F)** GO analysis results of the normal group between model groups; **(G)** GO analysis results of the WYHZF_H group between model group; **(H)** Key differentially expressed pathways.

Based on the previous confirmation that WYHZF can significantly improve DKD-related phenotypes and renal function in db/db mice, this study further employed renal transcriptome sequencing to decipher its molecular mechanism of action at the gene expression level. Four mice were randomly selected from each group, resulting in a total of 12 renal tissue samples from the control group, model group, and WYHZF_H group. Boxplots of normalized gene expression levels showed that the distribution of medians and interquartile ranges across all samples was highly consistent, with similar overall fluctuation ranges and no obvious abnormal outliers (Figure 5A). These findings indicated that the sequencing depth and normalization were satisfactory.

The results of PCA showed that the samples in the model group were significantly separated from those in the control group. In contrast, the WYHZF_H group was clearly distinguished from the model group and partially approached the control group, suggesting that WYHZF can significantly reshape the abnormal gene expression pattern (Figure 5B). Differential expression analysis revealed that compared with the control group, the model group had a total of 1,339 DEGs, including 722 upregulated genes and 617 downregulated genes (Figure 5C). Compared with the model group, the WYHZF_H group had 861 DEGs, with 577 upregulated and 284 downregulated, demonstrating that WYHZF exerts a significant regulatory effect on abnormal gene expression (Figure 5D) (Supplementary Table S4). Venn analysis further showed that a subset of DEGs overlapped between the model group and the WYHZF_H group. These genes exhibited abnormal changes in the model group but tended to be restored after WYHZF intervention, indicating that WYHZF has a reversal effect on key pathology-related genes (Figure 5E). GO enrichment analysis indicated that the DEGs of the model group compared with the control group were significantly enriched in pathways such as immune response, inflammatory response, extracellular region, and fatty acid and steroid metabolism, suggesting that immune activation and lipid metabolism disorders coexist during DKD progression (Figure 5F). In contrast, the DEGs after high-dose WYHZF intervention (vs. model group) were mainly enriched in terms including MHC class II complex, antigen processing and presentation, immunoglobulin complex, and regulation of immune response (Figures 5G,H). These findings underscore that WYHZF may exert renal protective effects by regulating MHC class II molecule-related biological processes. The results of KEGG analysis and related data are presented in Supplementary Figure S1 and Supplementary Tables S5,S6.

### 3.6 Prediction of molecular interactions between active metabolites and H2-Aa/H2-Ab1

Transcriptomic analysis showed that WYHZF extract exerted a significant impact on the diabetic kidney disease animal model, particularly by markedly altering the expression of H2-Aa and H2-Ab1. To further clarify these effects, we selected key absorbed metabolites for molecular docking and molecular dynamics simulations with H2-Aa and H2-Ab1, and the results of this part of the study provided an important reference for the subsequent WB validation.

We performed molecular docking of these 14 metabolites using H2-Aa and H2-Ab1 as receptors. The binding energy results indicated that, overall, aromatic phenolic acid and flavonoid metabolites exhibited stronger affinity for both proteins. Among them, Ononin, Catechin, (2Z)-6-hydroxy-2-[(4-hydroxy-3-methoxyphenyl)methylidene]-2,3-dihydro-1-benzofuran-3-one, 7-hydroxy-3-(4-methoxyphenyl)-4H-chromen-4-one, and Apigenin all showed binding energies ≤ −6.8 kcal/mol with H2-Aa, and their binding energies with H2-Ab1 also ranged from approximately −5.3 to −6.2 kcal/mol. These molecular interactions were significantly better than those of other metabolites (Table 2).

**TABLE 2:** Molecular docking scores of H2-Aa and H2Ab1 and potential blood-absorbed metabolites.

Metabolites	H2-Aa	H2Ab1
Ononin	−7.975	−6.215
Catechin	−7.189	−5.353
(2Z)-6-hydroxy-2-[(4-hydroxy-3-methoxyphenyl)methylidene]-2,3-dihydro-1-benzofuran-3-one	−7.052	−5.689
7-Hydroxy-3-(4-methoxyphenyl)-4H-chromen-4-one	−7.01	−5.911
Apigenin	−6.855	−5.653
Ferulic acid	−5.554	−4.478
2-Hydroxycinnamic acid	−5.277	−4.651
Gallic acid	−5.076	NA
Gentisic acid	−4.822	−4.307
2-Anisic acid	−4.776	−4.204
Phloroglucinol	−4.057	−3.395
Nipecotic acid	−3.983	−3.763
Glutaric acid	−3.799	−3.631
Succinic acid	−3.671	−3.59

**FIGURE 6:**
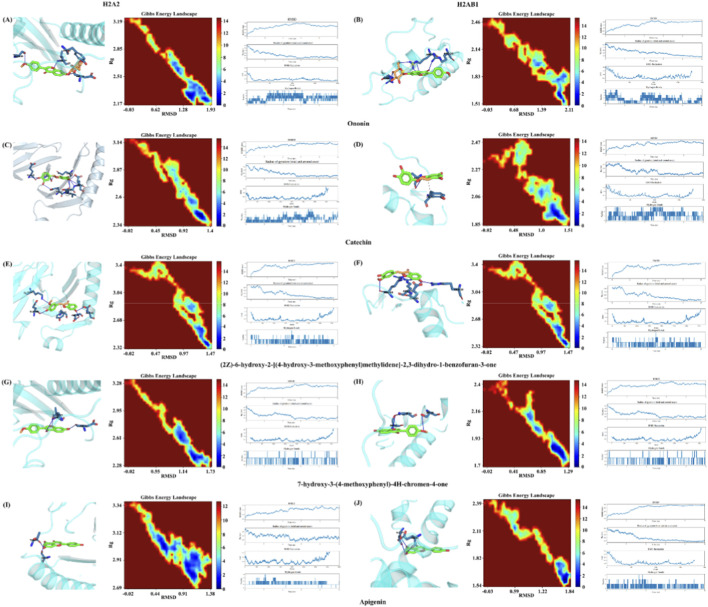
Molecular docking and molecular dynamics analysis between active metabolites of WYHZF extract and H2-Aa/H2-Ab1. **(A)** H2A2 and Ononin; **(B)** H2AB1 and Ononin; **(C)** H2A2 and Catechin; **(D)** H2AB1 and Catechin; **(E)** H2A2 and (2Z)-6-hydroxy-2-[(4-hydroxy-3-methoxyphenyl)methylidene]-2,3-dihydro-1-benzofuran-3-one; **(F)** H2AB1and (2Z)-6-hydroxy-2-[(4-hydroxy-3-methoxyphenyl)methylidene]-2,3-dihydro-1-benzofuran-3-one; **(G)**H2A2and7-hydroxy-3-(4-methoxyphenyl)-4H-chromen-4-one; **(H)** H2AB1 and 7-hydroxy-3-(4-methoxyphenyl)-4H-chromen-4-one; **(I)** H2A2 and Apigenin; **(J)** H2AB1 and Apigenin.

To evaluate the dynamic stability of these interactions, 10-ns molecular dynamics simulations were performed for each of the five protein-ligand complexes. As shown by the docking conformations (Figures 6A–J), all ligands were stably embedded in the binding pockets of H2-Aa/H2-Ab1 and formed compact interaction networks with surrounding residues through hydrogen bonding, hydrophobic contacts, and π–π stacking. The Gibbs free energy landscapes revealed relatively continuous and clustered low-energy basins in the RMSD-Rg space for all complexes, with no obvious scattered high-energy conformational states, indicating that the systems predominantly remained in energetically favorable conformations during the simulation. RMSD and Rg trajectories showed small fluctuations and rapidly reached plateaus, suggesting that both the protein backbone and the overall complexes were structurally stable. In addition, the number of hydrogen bonds fluctuated within a persistently present range over time, indicating that the ligands maintained relatively long-lasting hydrogen-bond interactions with H2-Aa and H2-Ab1.

Taken together, the molecular docking and molecular dynamics results suggest that several representative blood-absorbed metabolites from the WYHZF extract can plausibly fit within, and maintain stable interactions with, the predicted binding pockets of H2-Aa and H2-Ab1 under the simulated conditions. These computational observations provide hypothesis-generating molecular-level support for a potential association between WYHZF metabolites and MHC class II–related molecules, which warrants further experimental validation.

### 3.7 Verification of the effect of WYHZF on the expression of MHC class II molecules in renal tissues

**FIGURE 7:**
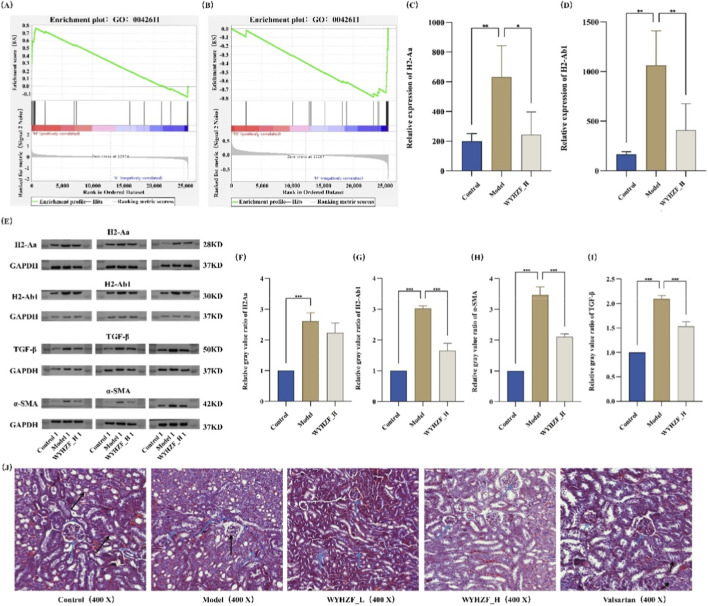
Effects of WYHZF on the renal expression of MHC Class II molecules. **(A)** GSEA analysis between control and model groups; **(B)** GSEA analysis between WYHZF_H and model groups; **(C)** Relative expression level of H2-Aa; **(D)** Relative expression level of H2-Ab1; **(E)** WB results of H2-Aa, H2-Ab1, TGF-β and α-SMA; **(F)** Relative grayscale value of H2-Aa; **(G)** Relative grayscale value of H2-Ab1; **(H)** Relative grayscale value of TGF-β; **(I)** Relative grayscale value of α-SMA; **(J)** Masson stain results. (Note: * for P < 0.05, ** for P < 0.01, and *** for P < 0.001).

GSEA was performed based on the renal transcriptome data, with the gene set related to the MHC protein complex selected for enrichment evaluation. The results showed that compared with the control group, the expression of MHC complex-related genes was significantly upregulated in the model group as a whole (Figure 7A). In contrast, after intervention with the WYHZF_H group, the enrichment score of this gene set decreased markedly, suggesting that WYHZF can inhibit the abnormally enhanced MHC class II-mediated immune activation in the context of diabetic kidney disease (Figure 7B).

Within this gene set, H2-Aa and H2-Ab1 were the two most prominently altered genes. Differential transcriptomic analysis revealed that compared with the normal group, both genes were significantly upregulated in the model group (P < 0.05); whereas after high-dose WYHZF intervention, their expression was markedly downregulated relative to the model group, with the overall expression levels trending toward those of the normal group (Figures 7C,D). Given that H2-Aa and H2-Ab1 encode the α-chain and β-chain of MHC class II molecules, respectively, their transcriptional levels can reflect changes in the local antigen-presenting capacity of the kidney. Further WB assays demonstrated that the changes in their protein expression were consistent with the transcriptomic results. The protein levels of H2-Aa and H2-Ab1 in renal tissues of the model group were significantly increased, whereas they were notably decreased following WYHZF treatment (Figure 7E; Supplementary Figure S3). Semi-quantitative analysis of band grayscale values also showed that the grayscale values of H2-Aa and H2-Ab1 in the model group were significantly higher than those in the normal group, while those in the WYHZF_H group were markedly lower than those in the model group (P < 0.05) (Figures 7F,G), which further verified the downregulatory effect of WYHZF on MHC class II-related molecules.

Given that persistent immune-inflammatory responses represent a critical upstream driver of renal fibrosis, we further examined the expression of TGF-β (a classic fibrotic pathway molecule) and α-SMA (a marker protein of myofibroblasts). WB results showed that TGF-β and α-SMA were markedly upregulated in the Model group, whereas their expression levels were reduced in the WYHZF group (Figure 6H; Supplementary Figure S4). This finding was further validated by the semi-quantitative analysis of band grayscale values (Figures 7I,J).

Masson’s trichrome staining of renal tissues revealed that the Model group exhibited increased collagen fiber deposition and aggravated interstitial fibrosis, while WYHZF intervention led to a reduction in the area of blue-stained collagen fibers. The therapeutic effect of the WYHZF_H group was comparable to that of the valsartan group (Figure 7K). Collectively, these results suggest that WYHZF may alleviate the degree of renal fibrosis by regulating MHC class II-related molecules, thereby exerting a multi-targeted renoprotective effect in diabetic kidney disease.

### 3.8 Effects of WYHZF on the gut microbiota in db/db mice

In addition to the direct effects of these active metabolites, the overall pharmacodynamic effects of these botanical drugs *in vivo* necessarily involve the complex interaction process of “metabolites -gut microbiota-host” (Yue et al., 2022). To clarify the overall functional advantages of WYHZF, this study employed 16S rRNA sequencing to detect the effects of WYHZF on the gut microbiota of DKD models, aiming to reveal another key pathway through which it exerts renal protective effects.

**FIGURE 8:**
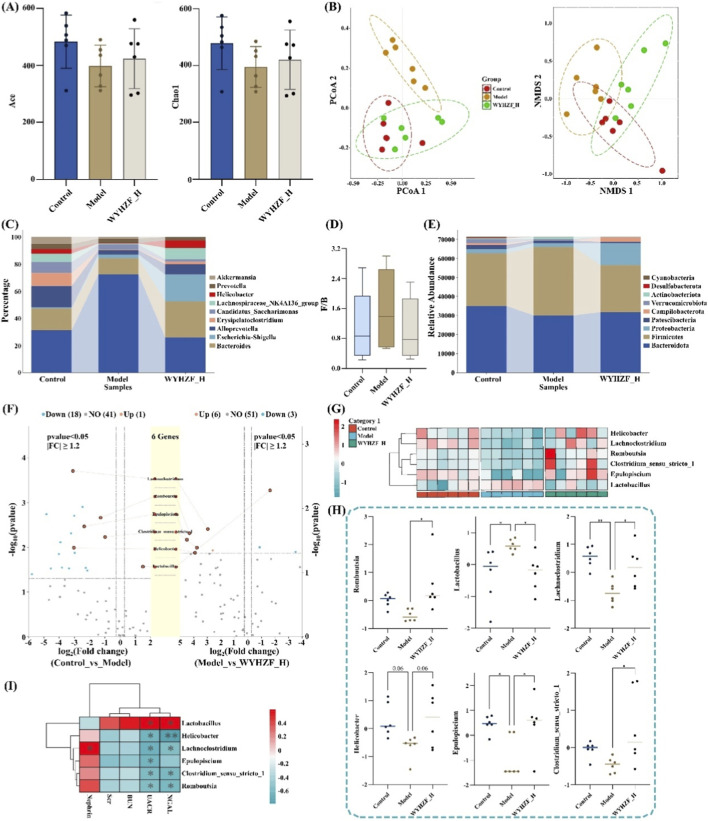
Effects of WYHZF on the gut microbiota in db/db mice **(A)** Alpha diversity analysis; **(B)** Beta diversity analysis; **(C)** Main taxonomic composition at phylum level; **(D)** Comparison of F/B ratio; **(E)** Main taxonomic composition at genus level; **(F)** Volcano plot for differential analysis; **(G)** Cluster heatmap; **(H)** Main differential microbiota at genus level; **(I)** Correlation analysis results. (Note: * for P < 0.05, ** for P < 0.01, and *** for P < 0.001).

α-diversity analysis based on Chao1 and Ace indices showed that compared with the control group, the gut microbiota species richness of the model group was significantly decreased. In contrast, after intervention with a high dose of WYHZF extract, the species richness exhibited a marked recovery trend (Figure 8A). For the β-diversity analysis, results from both NMDS and PCoA showed that the gut microbiota structure of the model group was significantly separated from that of the control group. The sample points of the WYHZF_H were distributed between the model group and the control group, and were closer to the control group than the model group (Figure 8B). These findings suggest that WYHZF intervention can restore the overall structure of gut microbiota in model mice.

Analysis at the phylum level revealed that Bacteroidota and Firmicutes were the dominant phyla (Figure 8C). The Firmicutes-to-Bacteroidota ratio (F/B ratio) is a key indicator for evaluating microbial community homeostasis and metabolic health. The results showed that WYHZF can effectively regulate the gut microbiota structure and rebalance the relative abundance between Firmicutes and Bacteroidota (Figure 8D). After removing low-abundance data, a total of 60 bacterial genera were identified at the genus level. The main dominant genera included *Lactobacillus* (43.17%), *Bacteroides* (18.27%), Alloprevotella (8.94%), and Erysipelatoclostridium (7.73%) (Figure 8E). Compared with control group, 18 genera were significantly downregulated and 1 genus was significantly upregulated in the DKD group (|FC| > 1.2, P < 0.05). After intervention with high-dose WYHZF, the abundances of the vast majority (45 out of 60) of genera were reversed, among which 5 genera were significantly upregulated and 1 genus was significantly downregulated (|FC| > 1.2, P < 0.05) (Figure 8F; Supplementary Table S7). Cluster analysis showed the abundance information of these 6 key differential genera (Figure 8G). The abundances of *Clostridium*_sensu_stricto_1, Epulopiscium, *Helicobacter*, Lachnoclostridium, and Romboutsia were significantly decreased in the DKD model, while their abundances were significantly restored after WYHZF intervention. In contrast, the abundance of *Lactobacillus* was significantly increased in the DKD model but significantly decreased in the WYHZF_H group (Figure 8H).

Based on Spearman’s correlation analysis, we found that the changes in the abundances of these six genera were significantly correlated with indicators such as UACR, NGAL and Nephrin (Figure 8I). In conclusion, WYHZF markedly ameliorated the overall structural disorder of the gut microbiota and regulated the abundances of key genera. This finding provides evidence supporting the role of WYHZF in ameliorating DKD via the “gut-kidney axis”.

## 4 Discussions

The occurrence and progression of DKD are recognized as the combined effects of multiple signaling pathways and molecular networks, and single-target drugs are often insufficient to achieve comprehensive control of disease progression (Tuttle et al., 2022). In recent years, studies on systems pharmacology and multi-omics have demonstrated that traditional botanical drugs, characterized by their multi-metabolite and network-based regulatory properties, are more suitable for managing complex diseases such as DKD (Shen et al., 2024; Gong, 2025). In this study, UPLC-QE-Orbitrap-MS was employed to identify the prototype and potential blood-absorbed metabolites of WYHZF, confirming that it is mainly composed of multiple classes of metabolites including phenolic acids, flavonoids/isoflavonoids, and triterpenoids. These metabolites have been reported in the literature to exert a variety of pharmacological effects such as anti-inflammatory, antioxidant, lipid-regulating, and pancreatic islet function-improving activities, which are consistent with the overall therapeutic effects of improving glycolipid metabolism and protecting renal function observed in our experiments (Liu et al., 2023; Ma et al., 2023). Further evidence at the network level verified that WYHZF extract not only extensively reverses DKD-related DEGs, but also simultaneously targets key MHC class II molecules such as H2-Aa/H2-Ab1 through multiple blood-circulating small molecules, thus exhibiting the typical characteristics of multi-metabolite and multi-targeted action.

Recent studies have demonstrated that the abnormal expression of MHC class II molecules in renal tubular and glomerular cells is one of the critical mechanisms underlying various types of renal injury and fibrosis (Zhou et al., 2021). MHC class II is involved in antigen presentation and CD4^+^ T-cell activation; its excessive activation can amplify local inflammatory responses and promote the production of profibrotic factors, thereby driving the progression of DKD. MHC class II genes such as H2-Aa and H2-Ab1 are markedly upregulated in renal tubules, which is closely correlated with renal function deterioration and inflammatory cell infiltration. In addition, mutations or deletions of MHC class II genes can significantly alter the immune microenvironment, indicating their pivotal role in maintaining immune homeostasis (Xu et al., 2022). Recently, there have also been reports that certain botanical drugs formulae alleviate diabetic renal fibrosis by regulating the expression of H2-Aa and H2-Ab1, which further underscores the potential value of these targets in DKD (Liang et al., 2024).

In the present study, both transcriptomic analysis and WB assays consistently demonstrated that the renal expression of H2-Aa and H2-Ab1 was significantly upregulated in the model group, whereas WYHZF extract reversed their overall expression pattern toward that of the normal group. This suggests that WYHZF extract may alleviate the renal immune-inflammatory burden at its source by attenuating MHC class II-mediated excessive antigen presentation and CD4^+^ T-cell activation. Notably, the present study also observed a favorable trend of improvement in TGF-β and α-SMA expression as well as Masson’s staining results. This indicates that while suppressing immune inflammation, WYHZF extract may also delay renal interstitial fibrosis via downstream profibrotic signaling networks, although this aspect requires validation through additional functional experiments.

**FIGURE 9:**
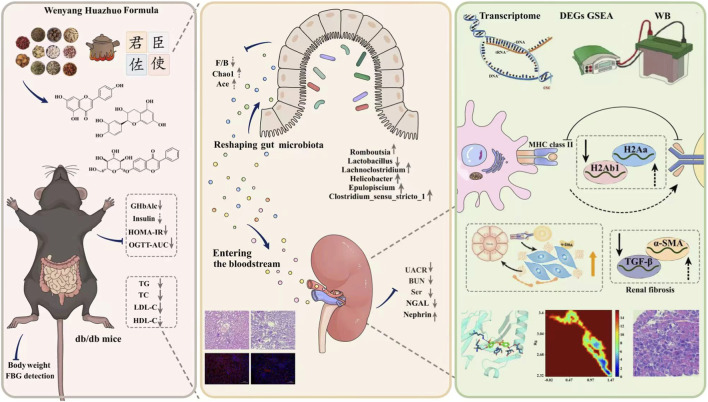
The schematic diagram of this study.

A growing body of evidence indicates that gut microbiota dysbiosis and abnormal production of their metabolites are among the crucial driving factors for the occurrence and progression of DKD (Lin et al., 2022). The gut microbiota can affect systemic inflammatory levels, oxidative stress, and energy metabolism via their metabolites, thereby exerting an impact on the kidneys; conversely, impaired renal function leads to the accumulation of uremic toxins, which in turn further disrupts microbiota homeostasis (Zhao et al., 2025). Multiple reviews have supported the notion that traditional Chinese medicine may ameliorate DKD by means of regulating microbiota structure and increasing SCFA-producing microbiota (Nie et al., 2019; Lin et al., 2022). In the present study, mice in the DKD model group exhibited decreased α-diversity, an increased F/B ratio, and abnormal abundances of multiple bacterial genera, which were highly consistent with previous report (Liu et al., 2025a). Intervention with high-dose WYHZF extract significantly restored the richness and diversity of the gut microbiota, corrected the abnormal F/B ratio, and regulated the abundances of multiple genera closely related to inflammation and energy metabolism, including *Clostridium* sensu stricto 1, *Helicobacter*, Lachnoclostridium, Romboutsia and *Lactobacillus*. Further results of correlation analysis revealed that the changes in these key genera were significantly correlated with renal injury indicators such as UACR, NGAL and Nephrin, suggesting that the modulation of gut microbiota by WYHZF extract contributes substantially to its therapeutic efficacy against DKD. Overall, elucidating the mechanism of action of these herbal compound formulas from the perspective of gut microbiota is a key approach to understanding their therapeutic efficacy, as well as a critical bridge connecting traditional theories with modern medical mechanisms (Cao et al., 2024; Gao, 2024; Liu et al., 2025b; Ning et al., 2024). Admittedly, in this study, we still lack crucial validation experiments to confirm the molecular mechanisms through which WYHZF improves DKD via gut microbiota. Future work will further explore key bacterial species and downstream molecular pathways by integrating methods such as fecal microbiota transplantation, metagenomics and molecular probe technique (Wang et al., 2026). The schematic diagram of this study is presented in Figure 9.

## 5 Conclusion

WYHZF extract could significantly ameliorate the glycolipid metabolic disorder in db/db mice, reduce levels of UACR, Scr and BUN, and alleviate the pathological damage of glomeruli and renal tubules, thereby exerting a potent effect on improving the progression of DKD. Further analyses integrating transcriptomics, WB, molecular docking and molecular dynamics demonstrated that WYHZF extract was capable of downregulating the abnormal activation of H2-Aa/H2-Ab1-related signaling pathways. Meanwhile, WYHZF extract could reshape the gut microbiota structure of DKD mice, restore microbiota diversity, correct the imbalance of key bacterial genera, which were closely correlated with renal function indicators. Overall, WYHZF extract fully embodies the advantages of botanical drug formulae characterized by multi-metabolite, multi-target and systematic regulation, providing robust experimental evidence for its further clinical application and the prevention and treatment of DKD.

## Data Availability

The data presented in the study are deposited in the NCBI repository, accession number PRJNA1432304.
