# Supplementary material for: Wenyang Huazhuo formula extract ameliorates diabetic kidney disease in db/db mice and is associated with modulation of MHC class II molecules and gut microbiota
Source: Front Pharmacol. 2026 Mar 31;17:1798497. doi: 10.3389/fphar.2026.1798497 (PMC13076160; doi:10.3389/fphar.2026.1798497)
Supplement: Supplementary file 1 [file Supplementaryfile1.docx]

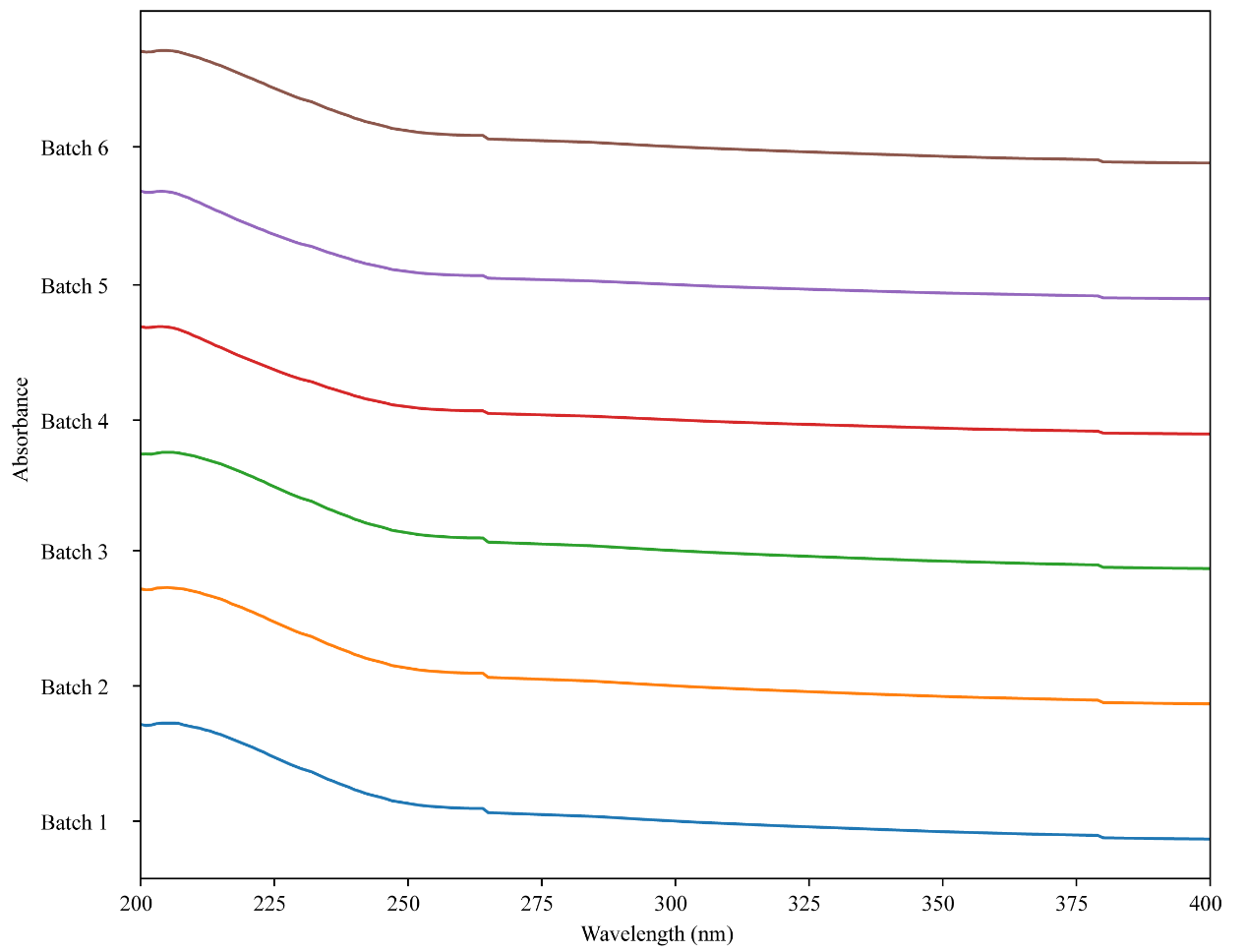


Figure S1: The UV-Vis fingerprint


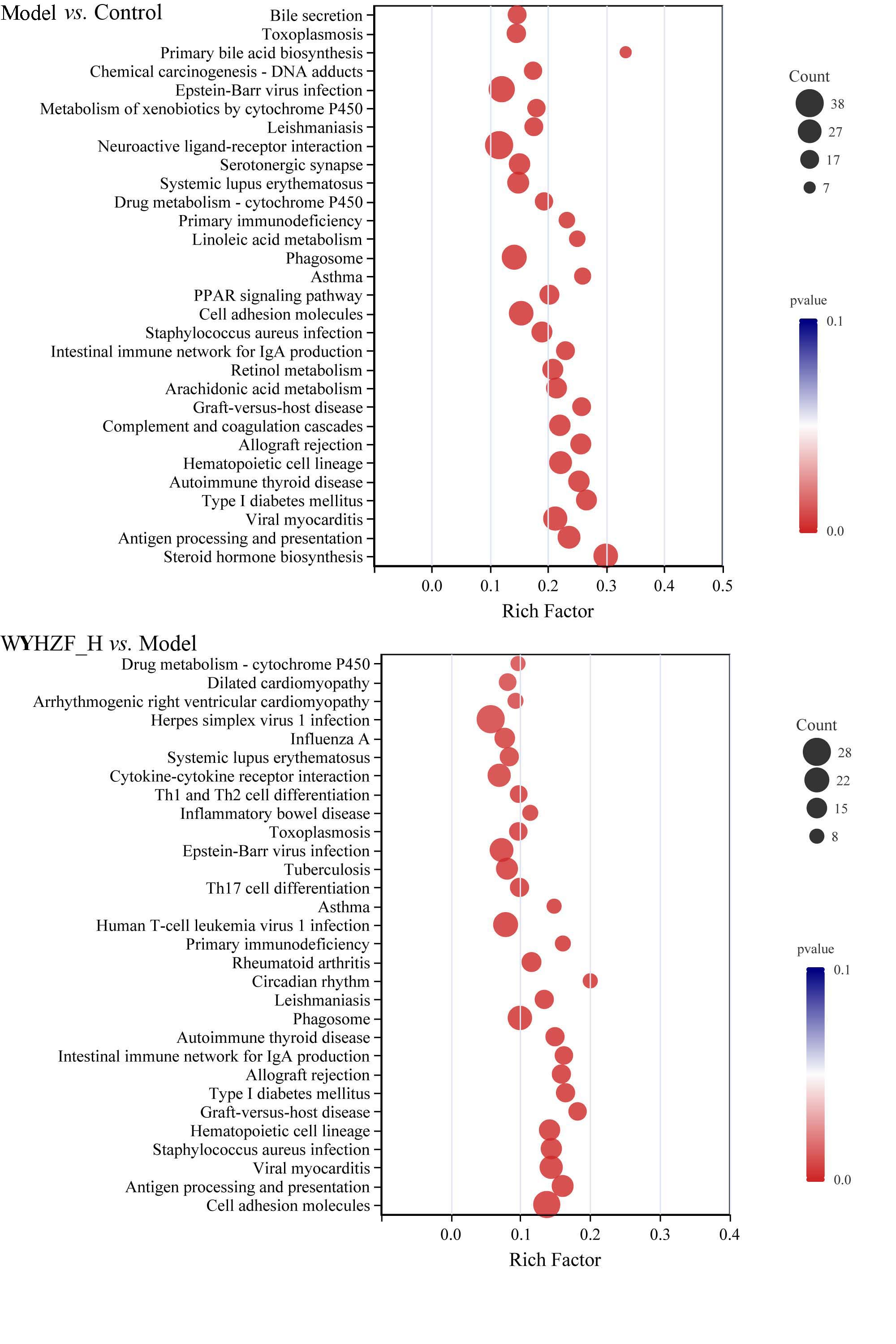


Figure S2: KEGG analysis based on transcriptome data


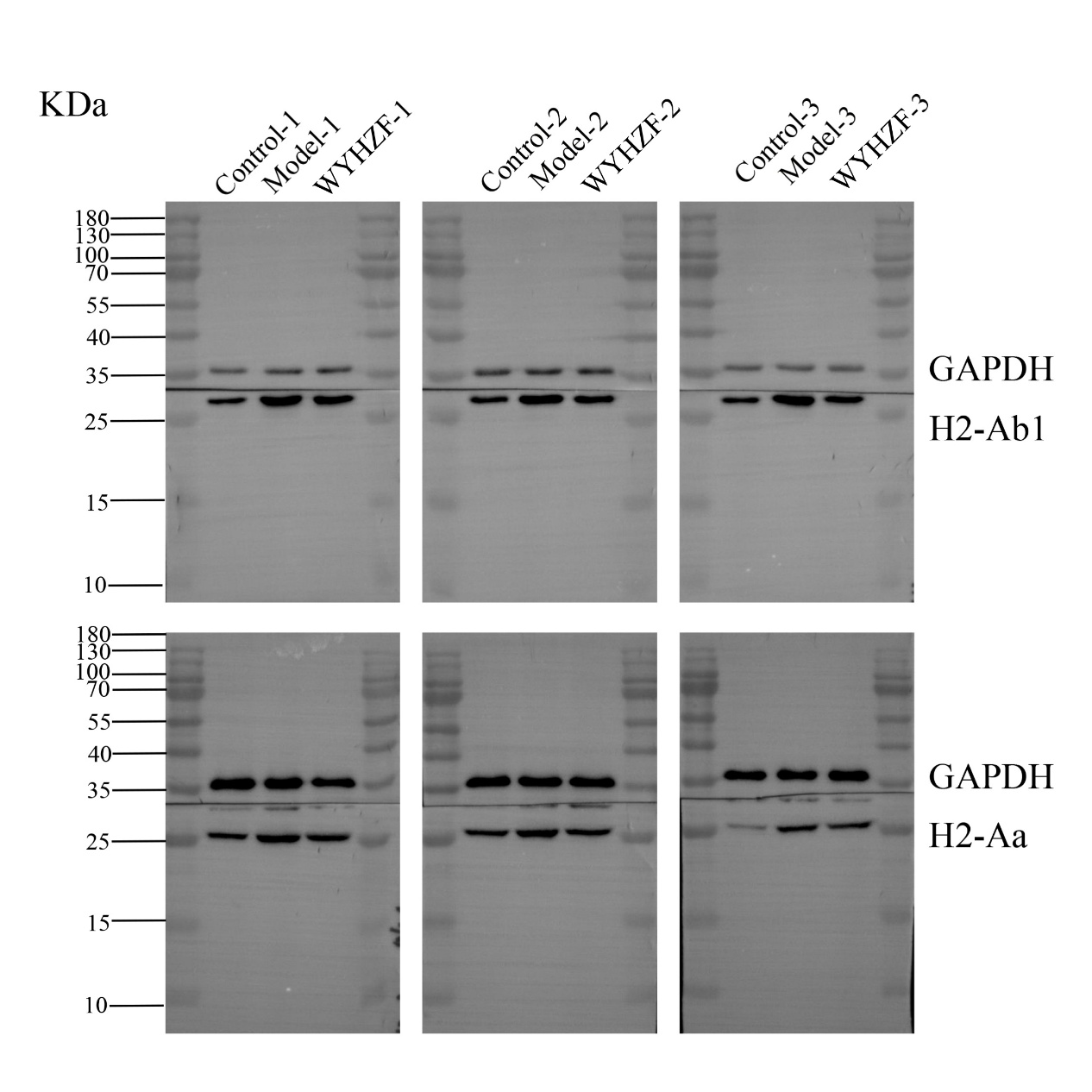


Figure S3. Western blot bands of H2-Ab1 and H2-Aa


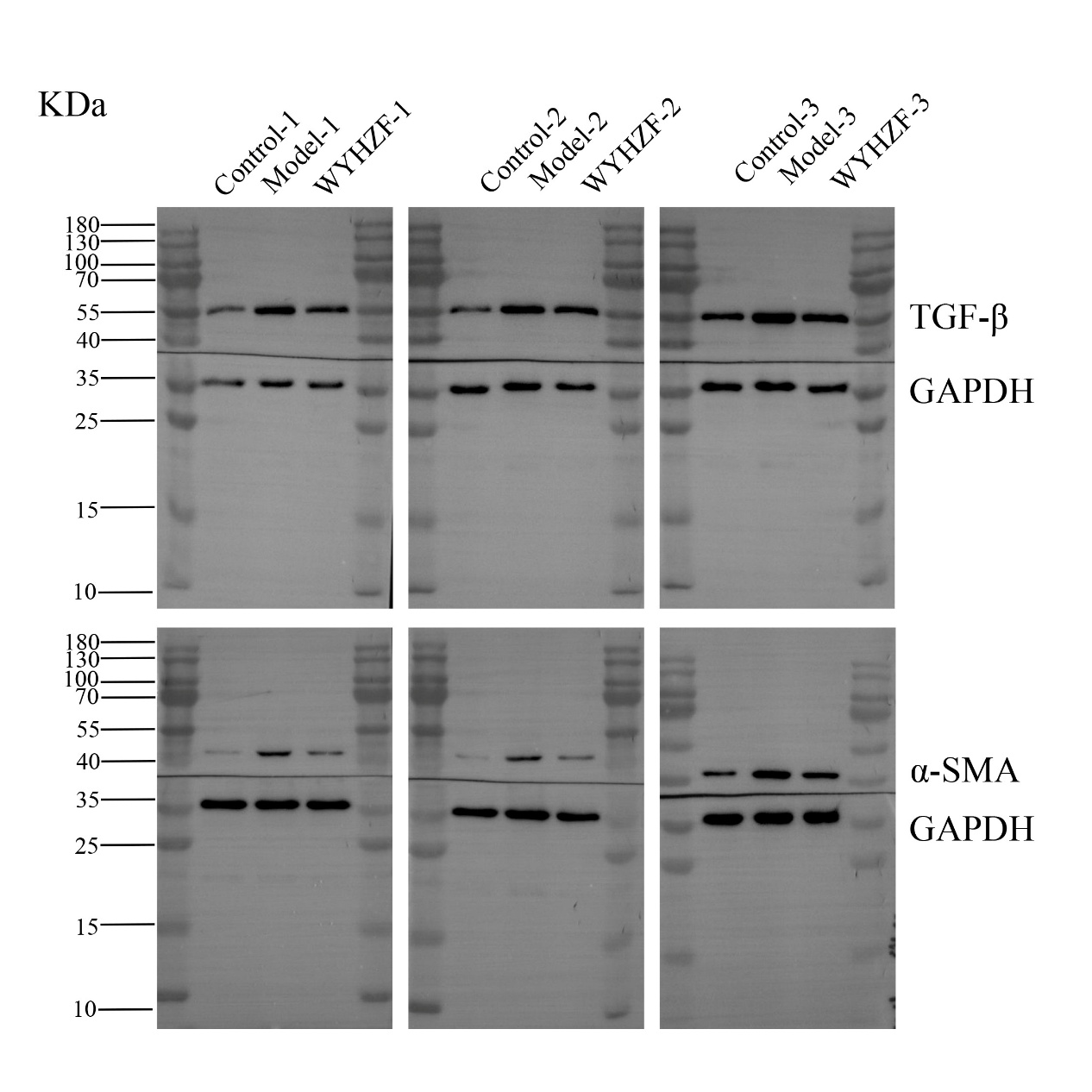


Figure S4. Western blot bands of TGF-β and α-SMA
